# Diagnostic value of microRNA-148/152 family in non-small-cell lung cancer (NSCLC): A systematic review and meta-analysis

**DOI:** 10.1097/MD.0000000000028061

**Published:** 2021-12-03

**Authors:** Long Cheng, Qinyun Li, Bangxian Tan, Daiyuan Ma, Guobo Du

**Affiliations:** a Department of Oncology, Affiliated Hospital of North Sichuan Medical College, Nanchong, China; b Department of Nephrology, Affiliated Hospital of North Sichuan Medical College, Nanchong, China.

**Keywords:** diagnostic value, meta analysis, microRNA-148/152, NSCLC

## Abstract

**Backgrounds:**

Non-small-cell lung cancer (NSCLC) is the most common type of lung cancer with extremely high morbidity and mortality.

**Objective:**

To evaluate the diagnostic value of the blood miR-148/152 family to NSCLC by meta-analysis.

**Methods:**

PubMed, Embase (via Ovid), The Cochrane Library, web of science, and Chinese National Knowledge Infrastructure were retrieved using miR-148, miR-152, and NSCLC as search terms for studies about miR-148/152 family in the diagnosis of NSCLC, the quality assessment of diagnostic accuracy studies was adopted to evaluate the quality of literature, STATA 12.0 and Meta-Disc 1.4 were used to conduct meta-analysis and to probe the clinical utility (with plotting the Fagan Nomogram).

**Results:**

A total 2145 cases in 8 trials published in 4 studies finally enrolled for final analysis. The area under the curve of the summary receiver operating characteristic was 0.87 [0.83–0.89], the pooled sensitivity was 0.79 [0.74, 0.83], the pooled specificity was 0.81 [0.76, 0.85] and the diagnosis odds ratio was 15.53 [10.88–22.17], the integrated positive likelihood ratio was 4.1 [3.30, 5.20] and the integrated negative likelihood ratio was 0.27 [0.22, 0.33].

**Conclusion:**

Current evidence indicated that miR-148/152 family might be served as novel non-invasive diagnostic biomarkers for NSCLC diagnosis with good sensitivity and specificity. it still needs more research with high quality, large sample sizes, and multiple centers for further verification.

## 1 Introduction

Lung cancer is a malignant tumor that has been a top cause of death within cancer for many years and it has extremely high morbidity and mortality worldwide. The epidemiological research showed that before the 2050s, lung cancer was still the main health issue confronted by the world public health.^[1]^ In the USA and part of European countries, the mortality of lung cancer shows a downtrend year by year.^[2,3]^ However, it still shows an uptrend in China.^[4]^ According to China's latest tumor epidemiology data reported by China's National Cancer Research Center in 2016, lung cancer is the leading cause of cancer death among both morbidity and mortality, indicating it became the heaviest burden of China's public health.^[4]^

Non-small-cell lung cancer (NSCLC) is the most common histological type that accounts for as high as 85% of lung cancer.^[5]^ Due to lack of specific symptoms, patients are always not diagnosed until the advanced stage and miss the best treatment opportunities such as surgical treatment, therefore, early diagnosis is crucial to reduce the high case fatality rate of lung cancer. In recent years, constant developments have been achieved in surgical treatment and traditional chemotherapy, however, prevention and control of lung cancer are still not optimistic, where the diagnostic technique is considered as one of the main reasons.^[6,7]^ It is regarded that some new invasive diagnosing techniques are currently available, including endoscopic ultrasound with fine-needle aspiration, transbronchial needle aspiration, transthoracic needle aspiration, and mediastinoscopy, thoracoscopy (video-assisted thoracoscopic surgery), enhance the accuracy of NSCLC diagnosis, each of these tests has particular risks and has technical considerations.^[8]^ So, the diagnosis and staging of lung cancer patients have more increasingly relied on minimally invasive tissue sampling.^[9]^ The need for non-invasive for the diagnosis of NSCLC has increased substantially over the recent years, as new non-invasive biomarkers, microRNA have been explored in the genomic diagnosis of NSCLC.

MiR-148/152 family is a series of miRNAs with the same seed sequence and similar biological function and mainly includes microrna-233a (mir-233a), microrna-233b (mir-233b), and microRNA-148/152 (miR-148/152).^[10]^ Several studies have reported that microRNA-148/152 has a certain diagnostic value for NSCLC, blood microRNA-148/152 is expressed differently in the blood of many NSCLC patients. However, most studies were conducted with a small sample size, and the observed associations were discordant. This research adopted a literature-based meta-analysis of eligible studies to evaluate the overall diagnostic value of the miR-148/152 family in NSCLC.

## 2 Methods

We systematically searched electronic databases, including PubMed, Embase (via Ovid), Cochrane library, Chinese National Knowledge Infrastructure, and Wan Fang Database. We searched using: “microRNA-148 OR hsa-mir-148 OR MIRN148b OR microRNA-148b OR miR-148a-5p OR miR-148a-3p OR MIRN148a OR miR-148a OR microRNA-152 OR hsa-mir-152 OR mir-152” and “Carcinoma, Non-Small Cell Lung OR Carcinomas, Non-Small-Cell Lung OR Lung Carcinoma, Non-Small-Cell OR Lung Carcinomas, Non-Small-Cell OR Non-Small-Cell Lung Carcinomas OR Non small Cell Lung Cancer OR Non-Small-Cell Lung Carcinoma OR Non Small Cell Lung Carcinoma OR Carcinoma, Non-Small Cell Lung OR Non-Small Cell Lung Cancer OR NSCLC” studies published up to 31 April 2021. Google Scholar, Baidu Scholar (Chinese) were also searched for any eligible studies. The reference lists of included studies were also manually searched to identify any relevant articles. Articles in English and Chinese were considered to be eligible.

### 2.1 Inclusion and exclusion criteria

The inclusion criteria of literature: (1) Published as complete article studied issues including expression of the miR-148/152 family and diagnostic value of NSCLC. (2) Objects of study: suspected confirmed NSCLC patients. (3) Diagnostic method: all NSCLC patients were diagnosed with NSCLC by the clinical gold standard. (4) Evaluating indicators: sensitivity and specificity of the miR-148/152 family for diagnosis of NSCLC and area under the curve (AUC), etc. (5) The studies had to provide sufficient information to construct the 2 × 2 contingency table,that is, false and true positives and negatives were provided.

Exclusion criteria: (1) The gold standard of diagnosis of NSCLC was not mentioned. (2) There's no receiver operating characteristic curve (ROC curve), single sensitivity, and specificity of the miR-148/152 family during diagnosis. (3) The literature could not be used because of repeated reporting and poor quality. (4) Review literature, abstracts, lectures, etc relenting to non-original research and basic research such as animal experiments.

### 2.2 Data extraction

For enrolled studies, the following information were extracted: the first author, the year of publishing, country, and sample sizes (including numbers of experimental groups and control groups), sample types, main experimental methods, the gold standard, the summary ROC curve (SROC), and the AUC, indicators such as sensitivity and specificity, numbers of true positive (TP), false positive (FP), false negative (FN), and true negative (TN) were recorded directly or calculated indirectly according to the original data of enrolled studies.

### 2.3 Quality assessment

To ensure the quality of meta-analysis, 2 investigators (LC and QY) evaluated the quality of enrolled studies according to quality assessment of diagnostic accuracy studies (QUADAS-2) provided by the Cochrane center. To avoid bias, when the 2 researchers had inconsistent evaluation opinions, a third investigator (GB) was consulted for further discussion to eliminate differences.

### 2.4 Statistical analysis

Literature quality evaluation was conducted by the QUADAS-2 tool^[11]^ of RevMan 5.3 (Cochrane Collaboration, Oxford, UK), meta-analysis was conducted by STATA 12.0 (Stata Corporation, College Station, TX, USA) and Meta-Disk for and testing heterogeneity caused by threshold effects by calculation of correlation coefficients of Spearman. Heterogeneity caused by other factors was mainly tested by *I*^2^ as a testing standard, *I*^2^ ≤ 50% indicated no statistical heterogeneity, and *I*^2^ > 50% indicated statistical heterogeneity, with Cochrane's *Q* statistic (α = 0.05) was also used for heterogeneity test. Random-effect models were applied for further analysis if heterogeneity was found. Otherwise, the random-effects model was used for calculating pooled sensitivity, specificity, positive predictive value, negative predictive value, and diagnostic odds ratios, the SROC curve was plotted and the AUC of SROC was calculated. By testing different pretest probability (25, 50, and 75), the Fagan nomogram was plotted based on Bayesian theory to explore clinical utility.^[12]^

## 3 Results

### 3.1 Data extraction

**Figure 1:**
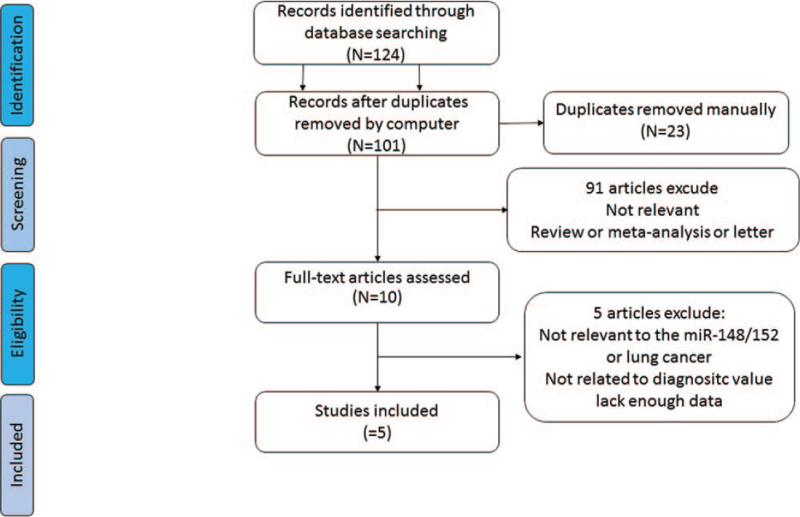
Flow chart of the study selection process.

A total of 124 potentially relevant citations, including reports in English and in Chinese (Table 1), were primarily retrieved after the initial database search, and 5 studies including 10 trials (4 studies in English and 1 study in Chinese were finally enrolled for final analysis (Fig. 1).^[13–17]^

**Table 1:** Characteristics of studies included in the present meta-analysis.

Number	Author	Year	Country	miR type	Patients/controls	Specimen	Detection method	TP	FP	TN	FN	Sensitivity (95% CI)	Specificity (95% CI)
1	Qiang et al	2016	china	miR-152	61/80	Serum	qRT-PCR	39	8	22	72	63.8	90.2
2	Li et al	2015	china	miR-148a	36/30	Serum	RT-PCR	28	4	8	16	77.8	80
3	Li et al	2015	china	miR-148b	36/30	Serum	RT-PCR	25	4	11	16	69.4	80
4	Li et al	2015	china	miR-152	36/30	Serum	RT-PCR	26	2	10	18	72.2	90
5	Dou et al	2015	china	miR-152	120/360	plasma	RT-PCR	103	67	17	293	86	81.3
6	Yang et al	2014	china	miR-148a	152/300	Serum	qRT-PCR	255	26	45	126	85	83%
7	Yang et al	2014	china	miR-148b	152/300	Serum	qRT-PCR	249	26	51	126	83	83
8	Yang et al	2014	china	miR-152	152/300	Serum	qRT-PCR	225	35	75	117	75	77
9	Abdollahi et al	2019	Iran	miR-148a-3p	43/43	Serum	qRT-PCR	35	17	8	26	82	77%
10	Abdollahi et al	2019	Iran	miR-152–3p	43/43	Serum	qRT-PCR	33	12	10	31	76	71

**Figure 2:**
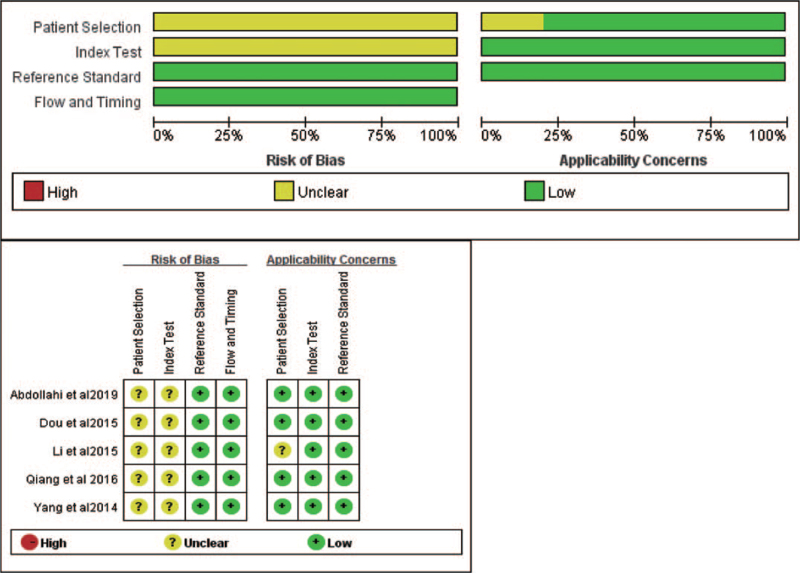
Quality of selected studies according to QUADAS-2 guidelines. QUADAS-2* *= the quality assessment of diagnostic accuracy studies.

With the utilization of the QUADAS-2 tool, the quality of enrolled studies was assessed as shown in Figure 2. We assessed the quality of diagnostic studies based on the QUADAS-2 criteria.^10^ It consists of 4 key domains: patient selection, index test, reference standard, flow and timing, and judge bias and applicability. Each is assessed in terms of risk of bias, and the first 3 domains were assessed with respect to applicability. Each item is answered with “yes,” “no,” or “unclear.” The answer of “yes” means low risk of bias, whereas “no” or “unclear” means the opposite.

### 3.2 Tests of heterogeneity and publication bias

**Figure 3:**
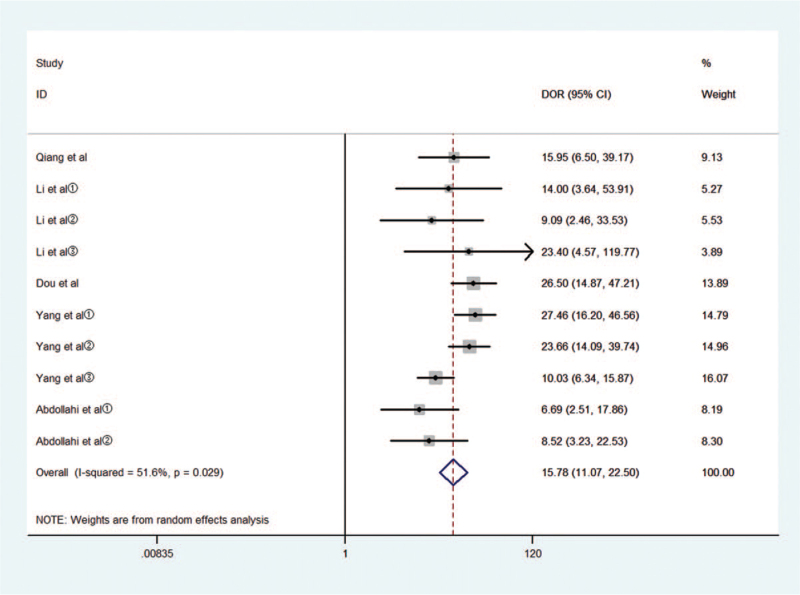
Diagnostic performance of miR-148/152 family and publication bias.

Threshold effects, considered as one of the important reasons for heterogeneity, were assessed by Spearman correlation coefficient with Meta-Disc software. The Spearman correlation coefficient was 0.396, *P* = .257 > .05, suggesting no obvious heterogeneity from the threshold effect. Cochran-*Q* is 18.58 in a diagnostic odds ratio, *I*^2^ = 51.6%, *P* = .029. Heterogeneity existed among the study designs caused by the non-threshold effect. The random-effects model was used to pool estimates (Fig. 3).

**Figure 4:**
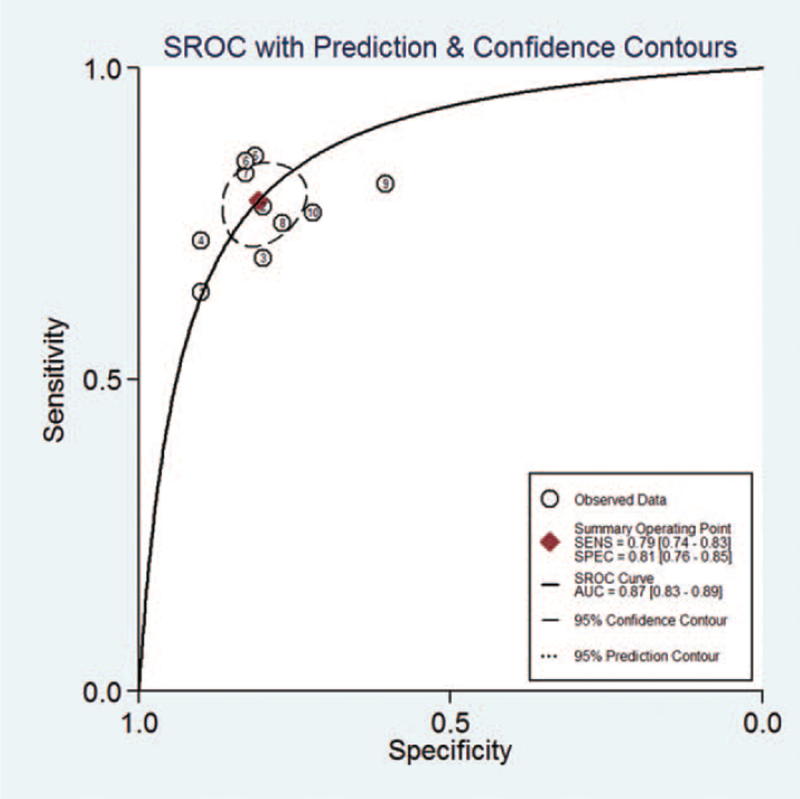
Summary receiver operating characteristic curve for miR-148/152 in the diagnosis of lung cancer.

**Figure 5:**
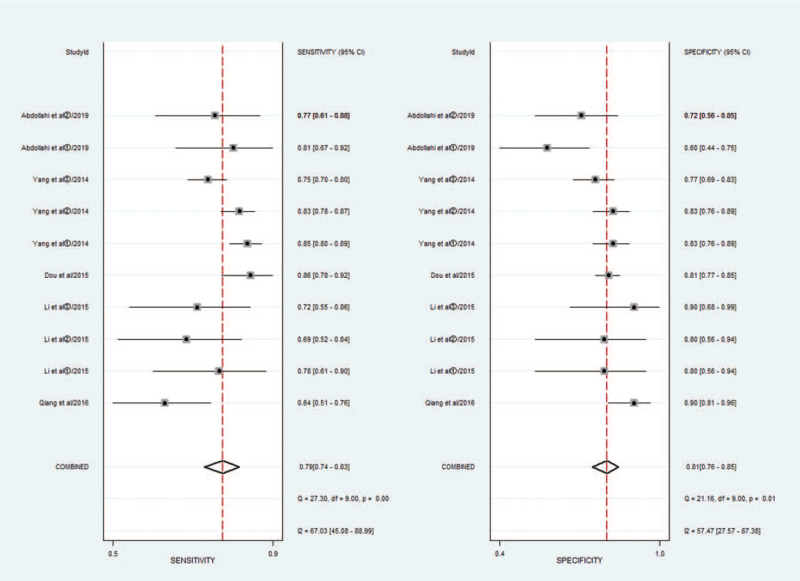
Forest plots of sensitivity and specificity for miR-148/152 in the diagnosis of lung cancer.

Meta-analysis was performed with the random-effects model, the pooled AUC of SROC of the miR-148/152 family used for diagnosis of NSCLC was 0.87 [0.83–0.89] (Fig. 4), *I*^2^ values for sensitivity and specificity were 67.03 (95% CI, 45.08–88.99) and 57.47 (95% CI, 27.57–83.78), respectively, suggesting mild significant heterogeneity. The pooled sensitivity and specificity was 0.79 [0.74, 0.83], 0.81 [0.76, 0.85], respectively (Fig. 5).

The pooled diagnosis odds ratio (DOR) was 15.53(95% CI, 10.88–22.17), the pooled positive likelihood ratio (PLR) was 4.1 [3.30, 5.20] and the pooled negative likelihood ratio (NLR) was 0.27 [0.22, 0.33].

**Figure 6:**
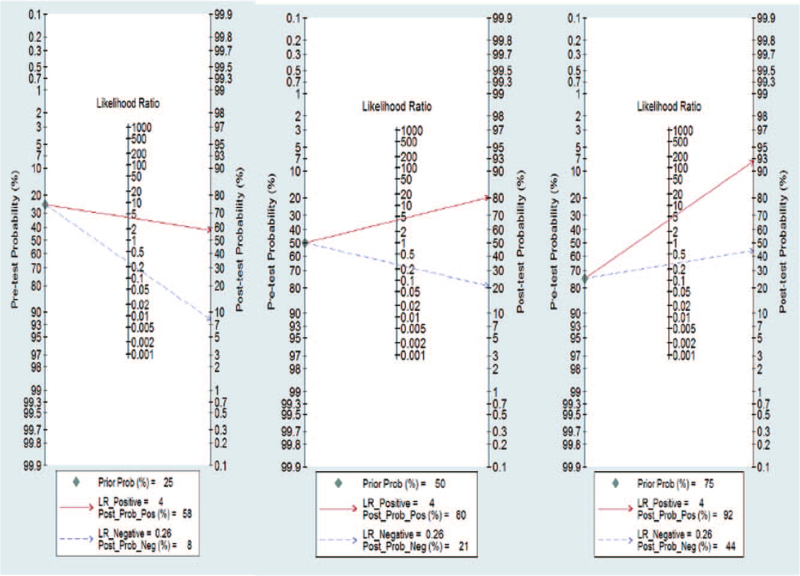
Fagan plots for miR-148/152 with 25%, 50%, and 75% pre-test probability of diagnosing NSCLC. NSCLC* *= non-small-cell lung cancer.

We utilized the likelihood ratios to simulate 3 clinical scenarios by implementing different pretest probabilities, 25% indicating relatively low clinical suspicion, 50% indicating moderate clinical suspicion, and 75% indicating a relatively high clinical suspicion. Using these likelihood ratios, the posttest probabilities on Fagan nomograms were calculated and plotted in Figure 6. With a pretest probability of 25%, the post probability positive (PPP) and post probability negative (PPN) were 58 and 8%, respectively. With a pretest probability of 50%, the PPP and PPN were 80% and 21%, respectively; and with a pretest probability of 75%, the PPP and PPN were 92% and 44%, respectively.

**Figure 7:**
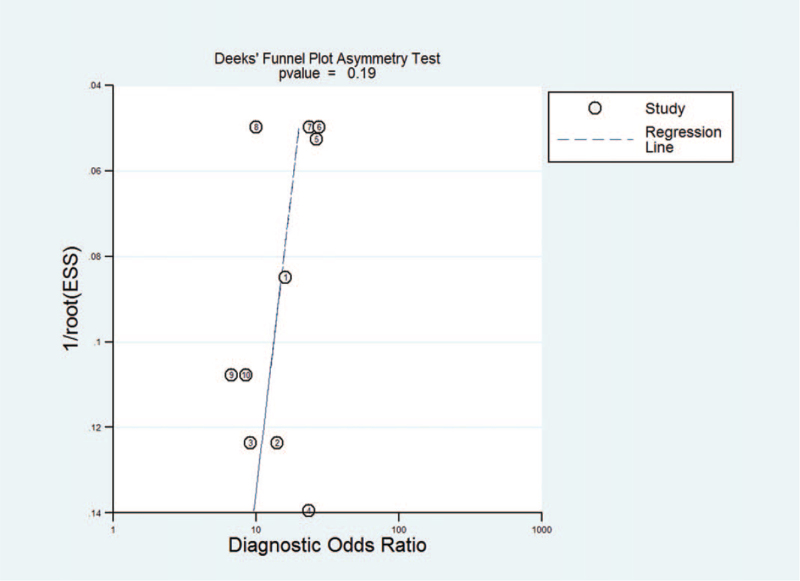
Deeks funnel plot asymmetry test for assessing publication bias.

The results of Deeks Funnel Plot Asymmetry Test showed no evidence of notable publication bias (*t* = −1.48, *P* = .19) (Fig. 7).

### 3.3 Sensitivity analysis and subgroup analyses

**Figure 8:**
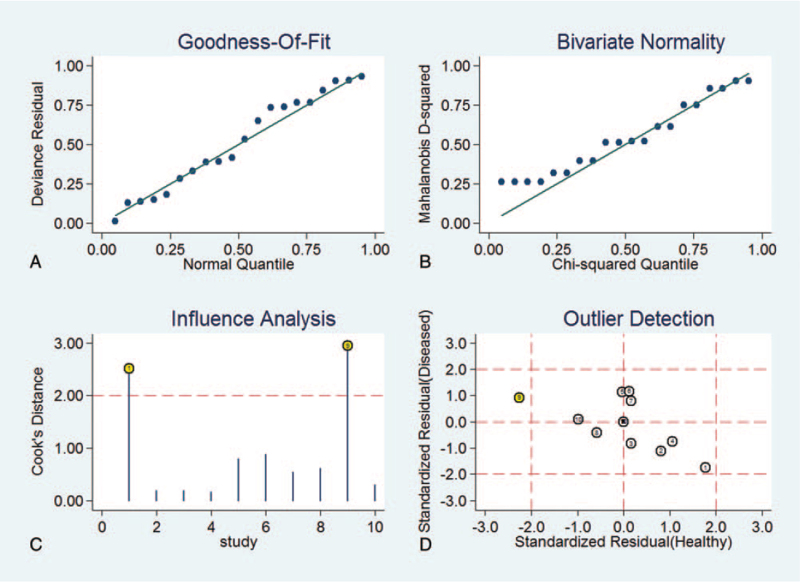
The results of sensitivity analysis.

The sensitivity analysis of the diagnostic value was shown in Figure 8. The goodness of fit and bivariate normality analyses confirmed that the selected analysis model was robust for the calculation of the pooled estimates. There were 2 deviated studies that may overshadow the robustness of the meta-analysis based on the influence analysis and outlier detection. After the exclusion studies, no significant changes in sensitivity (0.87 vs 0.86), specificity (0.80 vs 0.79), PLR (4.1 vs 4.0), NLR (0.26 vs 0.25), DOR (16 vs 16), and AUC (0.87 vs 0.86) were observed between the overall analysis with and without outlier. It is concluded that the meta-analysis of diagnostic value in the present study finding is robust.

Subgroup analysis was conducted based on microRNA type, sample type, and country. Table 2 shows none of the above covariates contributed to the heterogeneity (all *P* > .05). miR-148 has a similar accurate diagnostic value in comparison to the miR-152, with a sensitivity of 0.76 vs 0.82, specificity of 0.82 vs 0.78, and AUC of 0.86 vs 0.86. There are 9 Chinese studies and 1 study in Iranian. We analyzed the include data of china, sensitivity and specificity were 0.78 [0.73, 0.83], 0.82 [0.79, 0.85], respectively, PLR and NLR were 4.4 [3.8, 5.2], 0.26 [0.21, 0.33], respectively, DOR is 17 [12, 23], and AUC of 0.84 [0.81–0.87]. Subgroup analysis of different sample type, the sample type of serum in 9 studies while plasma in 1 study. However, there was no significant difference compared to our whole studies.

**Table 2:** Subgroup analyses of the included studies.

	Subgroup analyses	Number	Spearman correlation coefficient	DOR	Sensitivity (95% CI)	Specificity (95% CI)	PLR (95% CI)	NLR (95% CI)	DOR (95% CI)	AUC
type	miR-152	5	0.667/*P* = .219	Corchran-*Q* = 8.17 *P* = .0854	0.76 [0.68,0.82]	0.82 [0.76, 0.87]	4.2 [3.2, 5.6]	0.30 [0.23, 0.39]	14 [9,21]	0.86 [0.83–0.89]
	miR-148	5			0.82 [0.77,0.86]	0.78 [0.70,0.84]	3.7 [2.6,5.3]	0.23 [0.17,0.31]	16 [9, 29]	0.86 [0.83 –0.89]
country	china	9	0.170/*P* = .688	Corchran-*Q* = 12.81 *P* = .0768	0.78 [0.73, 0.83]	0.82 [0.79,0.85]	4.4 [3.8,5.2]	0.26 [0.21,0.33]	17 [12,23]	0.84 [0.81–0.87]
	Iran	1			/	/	/	/	/	/
Sample type	Serum	9	0.262/*P *= .496	Corchran-*Q* = 15.69 *P* = .0470	0.78 [0.73,0.82]	0.81 [0.75,0.85]	4.0 [3.1, 5.2]	0.28 [0.22,0.34]	14 [10, 21]	0.86 [0.83–0.89]
	Plasma	1			/	/	/	/	/	/

## 4 Discussion

The epidemiology of lung cancer in the majority of countries has consistently been reported and the situation remained to be not optimistic. Hence, it is quite important to use precise NSCLC inspection indexes that are proper for diagnosis or early screening of NSCLC. As classified into the same miRNA family due to the same seed sequence with approximately 6 to 7 nucleotides. In recent years, increasing evidence confirmed the aberrant expression of miR-148/152 family has been observed in tumor-like breast cancer,^[18]^ gastric cancer,^[19]^ hepatocellular carcinoma (HCC),^[20]^ and ovarian cancer.^[21]^

This is the first meta-analysis on the diagnostic utility of miR-148/152 family testing NSCLC patients, before analysis, we conducted a methodological quality assessment (Fig. 1) according to the QUADAS-2 analysis. Four studies of literature mentioned the gold standard of histopathological diagnosis that could distinguish NSCLC patients from healthy individuals effectively, while the study of Li et al mentioned the adoption of the NSCLC guideline of the American Joint Committee on Cancer, the also classified tumor node metastasis stages of patients, thus we considered a low risk of bias of Li's research of reference standard. For quality assessment of patient sampling, we found that description of patient sampling in all 5 studies was unclear; we also found that relevant research did not report that whether patients knew testing results of the gold standard before miRNA148/152 family tests, which was one of the biases of the research. During the flow and timing part of the quality assessment, we found that no literature mentioned assignment interval between miRNA148/152 family testing and the golden diagnosis. However, as this research was only about lung cancer patients, the length of intervals did not change the risks of lung cancer. As a result, we thought that time of miRNA148/152 family testing and intervals of the gold standard were low-risk events. Meanwhile, this research neither includes unpolished literature nor literature without calculation of TP, FP, TN, and FN, which might also be a bias source of this research.

With the utilization of meta-analysis, we found that the summary ROC of the miR-148/152 family for diagnosis of NSCLC was 0.87, its pooled sensitivity was 0.79 [0.74–0.83] and its pooled specificity was 0.81 [0.76–0.85], indicating that the miR-148/152 family could be a potential diagnostic index distinguishing NSCLC from non-NSCLC. Omission of any study did not alter the statistical significance of the results (data not shown). Therefore, the results of the sensitivity analysis suggested that the data in this meta-analysis were relatively robust.

In the sensitivity analysis, we deleted 2 trials that might lead to heterogeneity in the results, but no factors causing heterogeneity were found after the exclusion of the studies. After that, we performed subgroup analysis according to microRNA type, specimen source, and country, etc. The diagnostic sensitivity and specificity of miR-148 and miR-152 were similar. However, the DOR value of the sample from China was higher than the total data (17 vs 15). It is not clear whether there are differences in expression among ethnic groups, and it may be related to the lack of studies outside China.

It should be pointed out that heterogeneity existed in the pooled analysis of sensitivity (*I*^2^ = 67.03%), thus it is needed to further confirm the sensitivity of miR-148/152 family.

In addition, this research also analyzed the diagnostic value of the miR-148/152 family for NSCLC from the perspective of clinical utility. The Fagan nomogram is a commonly used tool for testing the clinical utility of diagnostic indicators by setting up different pretest probabilities. This research carried out the fitting of clinical scenarios through likelihood ratios and found that when pretest probability was set up to 50% (relative low clinical suspicion), the positive posttest probability increased to 80% and the negative posttest probability reduced to 21%; when pretest probability was set up to 75% (relative high clinical suspicion), the positive posttest probability increased to 92% and the negative posttest probability reduced to 44%, indicating the good clinical utility of the miR-148/152 family for diagnosis of NSCLC.

In conclusion, miRNA148/152 family, with a pooled sensitivity of 0.79 (need further evidence), specificity of 0.81 and AUC of SROC at 0.83, the pooled specificity was 0.81, can be novel non-invasive diagnostic biomarkers for NSCLC diagnosis with acceptable sensitivity and good specificity. it still needs more research with high quality, large sample sizes, and multiple centers for further verification.

Since this article is a meta-analysis, the data are collected from published articles and it is not necessary to provide ethical approval.

## Author contributions

**Conceptualization:** Qinyun Li.

**Data curation:** Long Cheng,Qinyun Li.

**Project administration:** Daiyuan Ma.

**Supervision:** Bangxian Tan.

**Visualization:** Qinyun Li, Guobo Du.

**Writing – review & editing:** Long Cheng, Qinyun Li.
